# A Multidimensional and Longitudinal Exploratory Study of the Stability of Pregnancy Contexts in the United States

**DOI:** 10.1089/whr.2024.0008

**Published:** 2024-03-12

**Authors:** Melissa A. Markowitz, Lisbet S. Lundsberg, Aileen M. Gariepy

**Affiliations:** ^1^ Department of Obstetrics, Gynecology and Reproductive Sciences, Yale School of Medicine, New Haven, Connecticut, USA.; ^2^ Department of Obstetrics and Gynecology, Weill Cornell Medicine and New York Presbyterian Hospital, New York, New York, USA.

**Keywords:** United States, pregnancy intention, unintended pregnancy, pregnancy context

## Abstract

**Objective:**

Evaluate the longitudinal stability of six pregnancy contexts, including intention, in a diverse cohort of individuals experiencing delivery, abortion, or miscarriage.

**Methods:**

We enrolled individuals 16–44 years of age with pregnancies <24 weeks gestation in this longitudinal study between June 2014 and June 2015 in four US urban clinics. We assessed six pregnancy contexts (intention, wantedness, planning, timing, desirability, and happiness) at enrollment and 3-month follow-up. We constructed three-level categorical measures for each context defined as favorable, ambivalent, or unfavorable. We used Wilcoxon sign tests to evaluate changes in paired observations between pregnancy context measures over time and by pregnancy outcome.

**Results:**

Among 121 participants at median gestational age of 7 weeks and 3 days, we found intention, wantedness, planning, timing, and happiness remained unchanged from enrollment in early pregnancy to 3-month follow-up. Individuals demonstrated changes in desirability; pregnancy assessments shifted toward less desirable from enrollment to follow-up (*p* = 0.01) (*i.e.,* desired to ambivalent, or ambivalent to undesired). Among participants choosing delivery (57%), assessments shifted toward more favorable planning (*i.e.,* unplanned to ambivalent, or ambivalent to planned) (*p* < 0.01), and less favorable desirability (*i.e.,* desired to ambivalent or ambivalent to undesired) (*p* < 0.01) at follow-up. Among participants choosing abortion (28%), assessments shifted toward more unfavorable planning (*i.e.,* planned to ambivalent, or ambivalent to unplanned) at follow-up (*p* < 0.01).

**Conclusion:**

In multidimensional, longitudinal assessment, pregnant participants' perspectives on five of six pregnancy contexts remained unchanged between enrollment and 3-month follow-up; only desirability shifted. Pregnancy planning perspectives differed by pregnancy outcome.

Human Research Subjects Protection Program: 1310012926.

## Introduction

Up to 45% of all pregnancies in the United States are considered unintended,^1–5^ and more specifically, 38% are considered poorly timed and 18% are considered unwanted.^6^ Reducing unintended or unwanted pregnancies is a major public health goal^5^ due to their association with adverse fetal, neonatal, and maternal outcomes, including higher rates of miscarriage, preterm birth, and maternal depression and anxiety.^7,8^ However, these associations are disputed because most measures of pregnancy intention are nonstandardized, binary, and subject to recall bias, as they are often assessed late in the third trimester or postpartum.^9–17^ For example, the National Survey of Family Growth (NSFG) and Pregnancy Risk Assessment Monitoring System (PRAMS) are standard for the assessment of pregnancy intention through in-person interviews (NSFG) or questionnaires given to postpartum women (PRAMS).^18,19^

These retrospective assessments of intention have been challenged as flawed, not only for their exclusion of pregnancies that result in miscarriage or abortion but also for assuming pregnancy intention to be a conscious decision, despite evidence of seemingly misaligned behaviors and pregnancy intention (*i.e.,* nonuse of contraception is not necessarily an indicator of pregnancy intention or planning).^20–22^ Studies on pregnancy intention similarly focus on pregnancy outcomes of only delivery^23,24^ or abortion^25–27^ and exclude miscarriage. In addition, previous studies conflate measures of desire^28^ and wantedness^26,29^ with pregnancy intention.

Previous studies of pregnancy intention are also criticized for assuming participants' perceptions of unintended pregnancy to be unchanged throughout pregnancy,^14,29^ a potential flaw that is further magnified by the fact that it is often measured retrospectively, sometimes up to 5 years after birth, and may be limited by recall bias.^19,30^ Prior longitudinal studies that have tried to assess stability of pregnancy intention are limited by retrospective assessments, focus on one birth outcome only (*e.g.,* delivery or abortion), and prolonged intervals between assessments ranging from 6 months to 7 years, lacking the granularity to assess changes in intention, while a person is still pregnant.^23,27,31^

As feelings about pregnancy may shift from preconception to postconception, or intrapartum to postpartum, retrospective assessment of pregnancy places an unrealistic onus on participants to recall how they felt at prior pivotal moments in the pregnancy. Recent publications challenge researchers to move beyond binary measures of intention, utilize more inclusive, multidimensional measures of pregnancy intention, include longitudinal measures to assess stability, and include pregnancies that end in abortion, miscarriage, and delivery.^10,13,17,32,33^

To begin addressing these challenges, we performed an exploratory longitudinal cohort study of pregnant people enrolled in early pregnancy and followed until pregnancy resolution (*i.e.,* abortion, delivery, miscarriage) at four urban clinics in the United States.^9,15,16,34–36^ We assessed six pregnancy contexts that are used to measure different components or aspects of unintended pregnancy, encapsulating both preconception perspectives (questions asking participants to assess their intention, wantedness, and planning *before* becoming pregnant) and postconception perspectives (questions asking participants to assess pregnancy timing, desirability, and happiness *now* that they are pregnant).^9^

Our previous analyses demonstrate the importance of using multidimensional pregnancy contexts because different pregnancy contexts are associated with different outcomes. For example, unplanned pregnancies were associated with low social support, while unintended pregnancies were not.^15^ To extend this body of research, next we performed a longitudinal assessment of each pregnancy context over time and investigated whether pregnancy contexts differed by pregnancy outcome (delivery, abortion, or miscarriage).

## Materials and Methods

### Study design

We evaluated data from the Experiencing PREgnancy, Sharing Stories Study,^9,15,16,34–36^ a longitudinal assessment of individuals presenting for walk-in pregnancy testing or abortion care at four clinical sites in New Haven, Connecticut, from June 2014 to June 2015. Eligible participants were pregnant individuals 15–44 years of age, <24 weeks gestational age by last menstrual period, and English or Spanish speaking. After providing written consent, participants completed a self-administered paper questionnaire within 1 week of a positive clinic pregnancy test or abortion care visit that assessed sociodemographic and participant characteristics, medical history, reproductive history, and six pregnancy contexts, as previously described (Supplementary Table S1).^9,34,36^ Participants completed the 3-month follow up through a phone survey (within 2 weeks of the 3-month follow-up date). We assessed pregnancy outcomes (delivery, abortion, or miscarriage) at 3-month telephone follow-up or through medical chart review. The institution's Human Research Protection Program approved this study.

### Measures of pregnancy context

We assessed six distinct pregnancy contexts at enrollment and 3-month follow up: Intention, wantedness, planning, timing, desirability, and happiness with pregnancy news.^9,15,16,34,36^ We have further categorized these into three preconception contexts (intention, wantedness, and planning), which asked participants to consider their perspectives about pregnancy *before* becoming pregnant, and three postconception contexts (timing, desirability, and happiness), which asked participants to consider their perspectives about pregnancy *now* that they are pregnant (Supplementary Table S1).

We assessed pregnancy intention, wantedness, and timing from individual questions from the London Measure of Unplanned Pregnancy (LMUP) and pregnancy planning from the composite LMUP score. The LMUP is a six-item measure that includes questions about intention, wantedness, timing, contraception use, partner communication about a potential pregnancy, and preparation (*i.e.,* use of folic acid, eating a healthy diet, cutting down on drinking alcohol) (Supplementary Table S2).^37^

Pregnancy planning was assessed using the LMUP by summing the six individual questions' responses, with each question assigned values from 0 to 2. We categorized LMUP scores as unplanned (scores 0–3), ambivalent (scores 4–9), and planned (scores 10–12).^37^ Because the composite LMUP score for planning includes components that may not actually be part of planning (*e.g.,* nonuse of contraception is given a score of “2” and is counted as an indication of planning), we analyzed its relevant individual components (intention, wantedness, and timing) as well as the LMUP's composite score for planning. Pregnancy desirability and happiness were assessed using stand-alone questions (Supplementary Table S1).

Consistent with prior publications on pregnancy context,^15,34–36^ we categorized each context into three-level categorical variables of favorable, ambivalent, and unfavorable (Supplementary Table S1). For example, we categorized intention as favorable (“I intended to get pregnant”), ambivalent (“My intentions kept changing), or unfavorable (“I did not intend to get pregnant”).

### Statistical analysis

We used descriptive statistics to analyze participant characteristics and pregnancy contexts at enrollment and 3-month follow-up. We utilized Wilcoxon sign tests for paired data using a three-point scale to evaluate changes between pregnancy context at enrollment versus 3-month follow up. The paired data analysis allowed individual responses to pregnancy contexts at enrollment to be directly aligned with each participant's response at follow-up. We also assessed change in context measures stratified by pregnancy outcome (delivery, abortion, and miscarriage) using Wilcoxon tests. We graphed paired longitudinal data using Sankey diagrams, a flow algorithm previously used in longitudinal gynecologic studies assessing individual's decision-making and symptom assessment.^38,39^ Statistical analysis was conducted using SAS 9.4 (SAS Institute, Cary, NC).

## Results

### Study sample characteristics

Research staff screened a total of 361 individuals, of which 269 were deemed eligible and 161 completed enrollment. Our final analytic sample consisted of 121 participants, based on individuals who completed both enrollment and 3-month follow-up assessments. Among the 121 participants, 120 had known pregnancy outcomes. Participants ranged in age from 16 to 44 years with a median age of 26 years (Table 1). Median gestational age at enrollment was 7 weeks and 3 days. Seventy-one percent of participants completed the study in English, with the remaining 29% completing it in Spanish. Forty-five percent of participants self-identified as Hispanic, 34% as Black non-Hispanic, 13% as White non-Hispanic, and 8% as multiracial. Overall, 57% of pregnancies resulted in delivery, 28% in abortion, and 15% in miscarriage.

**Table 1.** Participant Characteristics and Sociodemographics, *n* = 121^a^

Characteristic	***n*** (%)
Age: median (IQR)	26 (22–32)
Gestational age at enrollment: median (range)	7.4 (4.0–23.0)
Language study completed in	
English	86 (71.1)
Spanish	35 (28.9)
Race-ethnicity	
Black, non-Hispanic	40 (33.6)
White, non-Hispanic	16 (13.4)
Hispanic	54 (45.4)
Multiracial, Other	9 (7.6)
Education	
12 years/GED or less	74 (61.7)
Some college, college degree	46 (38.3)
Employment	
Unemployed/homemaker	68 (56.7)
Full time/part time	52 (43.3)
Relationship status	
Single	43 (35.8)
Married	20 (16.7)
Living with partner, not married	43 (35.8)
Separated, divorced, widowed	14 (11.7)
Chronic medical problem	25 (20.7)
Ever diagnosed with depression	26 (21.5)
Ever diagnosed with anxiety	25 (20.7)
Parity	
0	30 (25.2)
1	43 (36.1)
2+	46 (38.7)
Previous miscarriage	41 (36.6)
Previous abortion	44 (38.6)
Smoking/tobacco products in past 3 months	38 (32.4)
Drinking alcohol past 3 months	66 (54.5)
Marijuana use past 3 months	22 (18.2)
What do you think you will most likely do?	
Plan to parent	77 (64.2)
Plan for adoption	2 (1.7)
Have an abortion	28 (23.3)
Don't know	13 (10.8)
Outcome of pregnancy	
Abortion	34 (28.3)
Delivery	68 (56.7)
Miscarriage	18 (15.0)

^a^ Total participants per question may not add to 121 due to missing observations.

GED, general educational development; IQR, interquartile range.

### Longitudinal assessment of categorical pregnancy contexts over a 3-month period

#### Preconception contexts

**FIG. 1.**
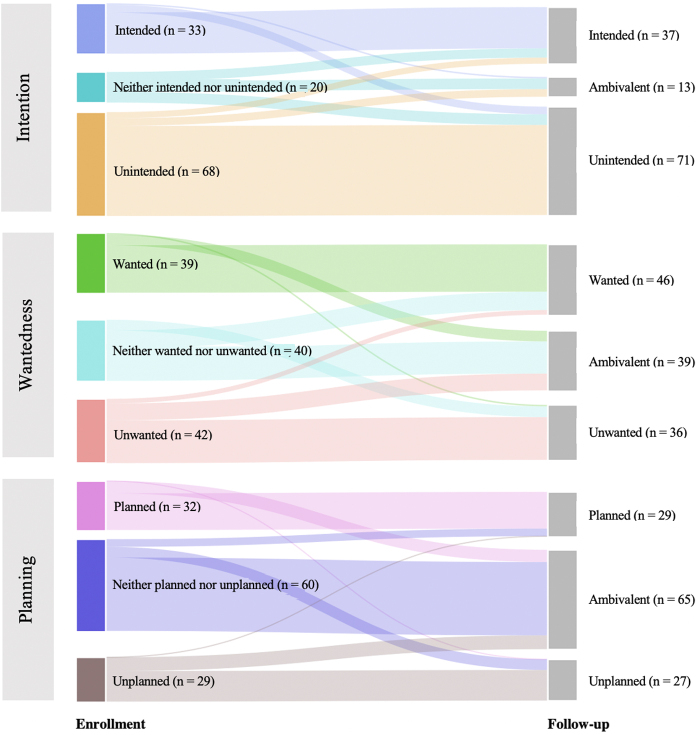
Preconception pregnancy contexts: Paired data comparing enrollment to 3-month follow-up, *n* = 121.

Examining preconception contexts at enrollment, most participants reported unintended pregnancies (56.2%) and half reported ambivalence about planning (49.6%). We found evenly distributed responses to wantedness at enrollment (34.7% unwanted, 33.1% ambivalent, and 32.2% wanted) (Table 2). We found no significant change in a paired analysis of the three preconception pregnancy contexts from enrollment to follow-up (Fig. 1).

**Table 2.** Pregnancy Contexts at Enrollment and Follow-Up, *n* = 121

Pregnancy context	Enrollment responses, ***n*** (%)			Follow-up responses, ***n*** (%)			***p*** ^a^
	Favorable	Ambivalent	Unfavorable	Favorable	Ambivalent	Unfavorable	
Preconception							
Intention	33 (27.3)	20 (16.5)	68 (56.2)	37 (30.6)	20 (16.5)	71 (58.7)	0.85
Wantedness	39 (32.2)	40 (33.1)	42 (34.7)	46 (38.0)	40 (33.1)	36 (29.8)	0.12
Planning	32 (26.4)	60 (49.6)	29 (24.0)	29 (24.0)	60 (49.6)	27 (22.3)	>0.99
Postconception							
Timing	45 (37.2)	45 (37.2)	31 (25.6)	42 (34.7)	45 (37.2)	29 (24.0)	>0.99
Desirability	62 (51.2)	26 (21.5)	33 (27.3)	47 (38.9)	26 (21.5)	39 (32.2)	0.01
Happiness	79 (65.3)	26 (21.5)	16 (13.2)	73 (60.4)	26 (21.5)	24 (19.8)	0.07

^a^ *p*-Values for Wilcoxon sign tests for nonparametric paired data by pregnancy context.

#### Postconception contexts

**FIG. 2.**
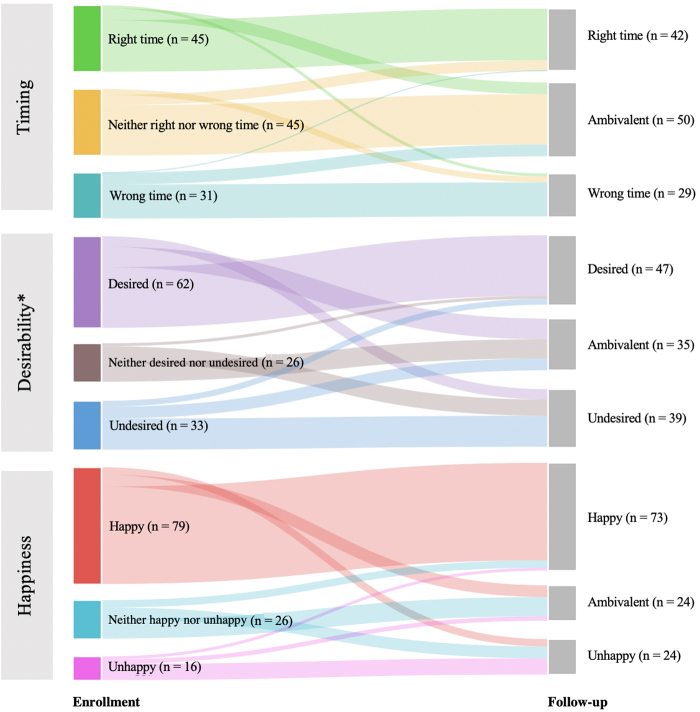
Postconception pregnancy contexts: Paired data comparing enrollment to 3-month follow-up, *n* = 121. *p < 0.05.

Examining postconception contexts at enrollment, most participants reported desired pregnancies (51%) and happiness with their pregnancies (51%). We found evenly distributed responses to timing at enrollment (37% at the right time, 37% ambivalent, and 26% at the wrong time). We found a significant change in participants' perspectives of desirability from enrollment to follow-up (*p* = 0.01) shifting toward less desired (*i.e.,* desired to ambivalent or undesired, or ambivalent to undesired) (Fig. 2). We found no significant change in the paired analyses for pregnancy timing or happiness from enrollment to follow-up.

### Stability of pregnancy contexts by pregnancy outcome (delivery, abortion, and miscarriage)

#### Delivery

##### Preconception contexts

**FIG. 3.**
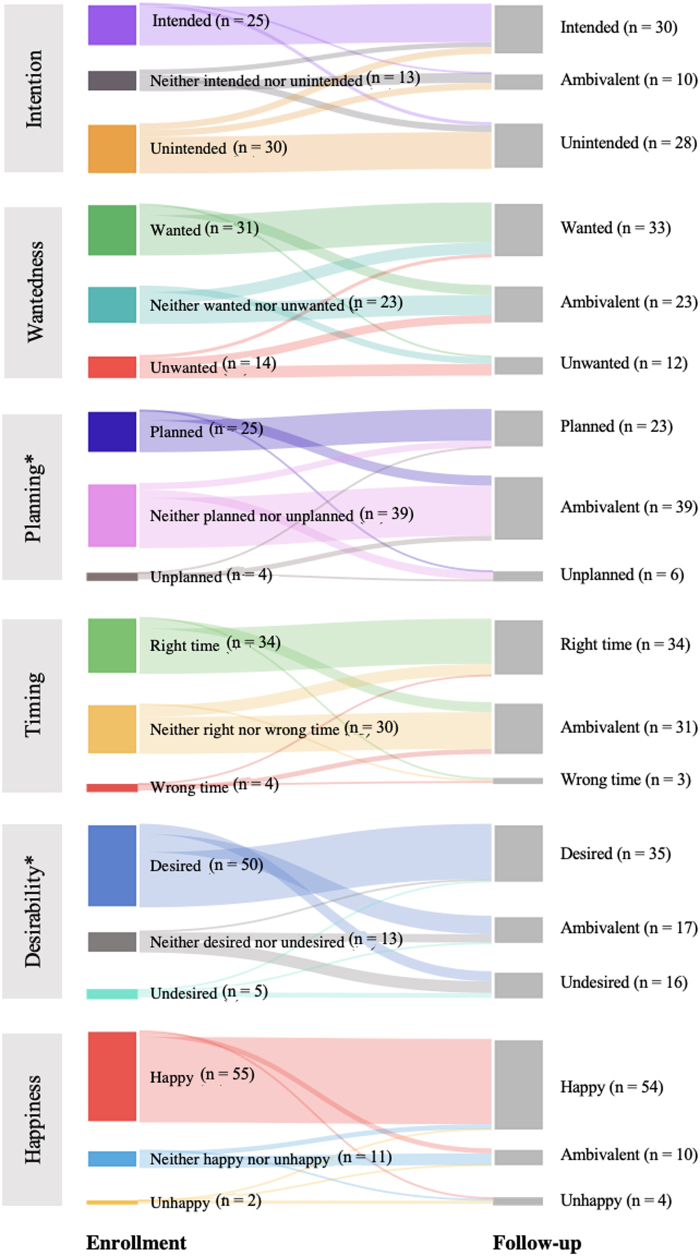
Participants undergoing delivery: Paired data comparing enrollment to 3-month follow-up, *n* = 68. *p < 0.01.

Among the 68 participants who had a delivery, a majority reported pregnancies that were ambivalently planned (57.4%) and just under half were wanted (45.6%) and unintended (44.1%) at enrollment (Supplementary Table S3). We found a significant change in participants' assessment of pregnancy planning from enrollment to follow-up (*p* < 0.01) shifting toward describing pregnancy planning as more favorable (*i.e.,* unplanned to ambivalent or planned, or ambivalent to planned) (Fig. 3). We found no change in the paired analyses for pregnancy wantedness or intention.

##### Postconception contexts

At enrollment, most participants who ultimately had a delivery reported pregnancies as desired (73.5%) and that they felt happy about (80.9%), while half were described as occurring at the right time (50.0%) (Supplementary Table S2). We found a significant change from enrollment to follow-up in participants' assessment of pregnancy desirability (*p* < 0.01) (Fig. 3) shifting toward less favorable (*i.e.,* desired to ambivalent or undesired, or ambivalent to undesired). We found no change in paired analyses for pregnancy happiness or timing.

#### Abortion

##### Preconception contexts

**FIG. 4.**
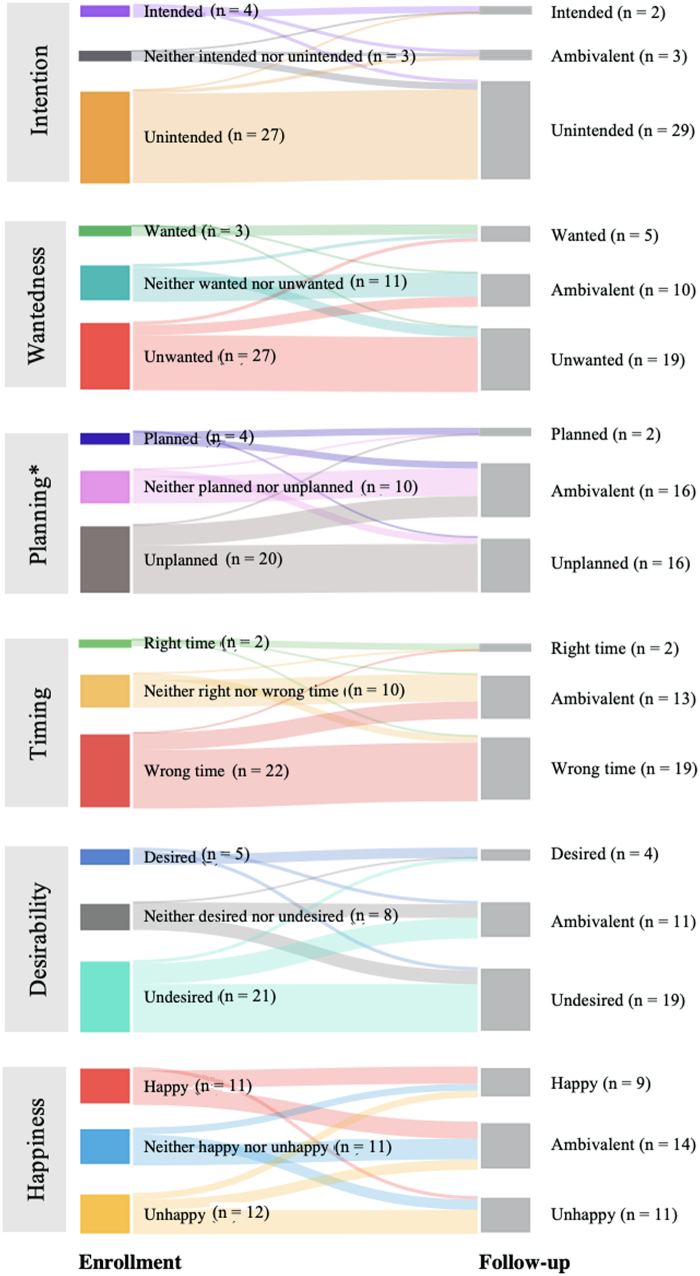
Participants undergoing abortion: Paired data comparing enrollment to 3-month follow-up, *n* = 34. *p < 0.01.

Among the 34 participants who had an abortion, a majority reported pregnancies that were unintended (79.4%), unwanted (58.8%), and unplanned (58.8%) at enrollment (Supplementary Table S4). We found a significant change in paired analysis of planning from enrollment to follow-up (*p* < 0.01) (Fig. 4). Individual perspectives on planning shifted toward less favorable (*i.e.,* planned to ambivalent or unplanned, or ambivalent to unplanned). No change was seen in paired analyses for pregnancy intention or wantedness.

##### Postconception contexts

At enrollment, most participants who ultimately had an abortion reported pregnancies that occurred at the wrong time (64.7%) and were undesired (61.8%) (Supplementary Table S3). Happiness was evenly distributed (32.4% happy, 35.3% unhappy, and 32.4% neither happy nor unhappy). We found no significant change in paired analyses for all postconception contexts.

#### Miscarriage

##### Preconception contexts

Among the 18 participants who experienced miscarriage, a majority reported the pregnancy as unintended (61.1%) or ambivalently planned (61.1%), and under half were unwanted (44.4%) at enrollment (Supplementary Table S5). We found no significant change in paired analyses for all preconception contexts.

##### Postconception contexts

**FIG. 5.**
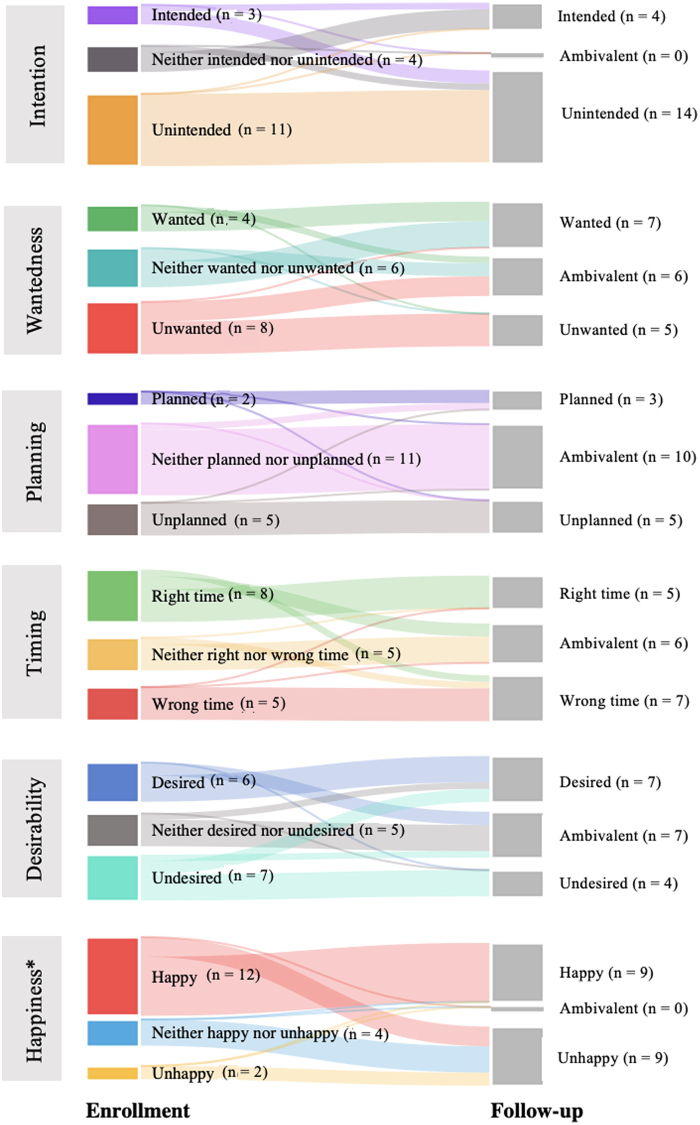
Participants undergoing miscarriage: Paired data comparing enrollment to 3-month follow-up, *n* = 18. *p < 0.05.

At enrollment, most participants who ultimately had a miscarriage reported happiness associated with the pregnancy (66.7%) (Supplementary Table S5). An even distribution was seen in pregnancy desirability (38.9% undesired, 33.3% desired, and 27.8% ambivalent) and timing (44.4% at the right time, 27.8% at the wrong time, and 27.8% ambivalent). We found a significant change in paired analysis of happiness from enrollment to follow-up among participants who experienced miscarriage (*p* = 0.02) (Fig. 5). Individual assessment of pregnancy happiness shifted toward less favorable (*i.e.,* happy to unhappy or ambivalent, or ambivalent to unhappy). We found no change in paired analyses for pregnancy desirability or timing.

## Discussion

In this longitudinal exploratory assessment of the stability of six pregnancy contexts among a diverse sample, we found that participants' perspectives on five of six pregnancy contexts remained unchanged from measurement in early pregnancy compared to 3-month follow-up. The overall lack of change in individual perspectives on pregnancy intention, wantedness, planning, timing, and happiness emphasizes the benefit of longitudinal, early pregnancy context assessment, in contrast to limited retrospective measures. Our findings of stability in pregnant individuals' responses to pregnancy planning, intention, and wantedness support reproductive choice in early pregnancy, including timely, individualized decisions for abortion or prenatal care.

Interestingly, among the whole cohort, we found a shift from enrollment to 3-month follow-up in participants' perspectives on pregnancy desirability (“Is this pregnancy desired?”), a postconception pregnancy context that asked individuals to consider their perspectives now that they are pregnant. Overall, participants were more likely to report pregnancies as undesired at 3-month follow-up compared to enrollment. However, this shift in desirability differed by pregnancy outcome. Participants who chose delivery demonstrated a significant shift in pregnancy assessment toward less desired (from desired to ambivalent or undesired, or ambivalent to undesired) at follow-up, a finding that was not seen in those who had an abortion or miscarriage. It is not clear why those choosing delivery reported a shift in desirability.

One possible explanation may be the shifting influence of social desirability bias, a bias toward answering questions about a pregnancy in a way that is socially acceptable.^24^ For example, the general excitement of a new pregnancy, which is often greeted with congratulatory remarks from family and friends, may influence participants toward desirability. As the pregnancy continues, key life circumstances may change such as a loss of partner or job, worsening economic situation, or unstable housing, and may impact the social acceptability of the pregnancy and participants' perspectives on the question of whether this pregnancy was desired. Of note, enrollment data were collected in-person through self-administered questionnaires and 3-month follow-up data were obtained through telephone interviews, and may also contribute to this shift in desirability.

In our exploratory longitudinal analysis of pregnancy contexts by different pregnancy outcomes (delivery, abortion, and miscarriage), we found additional variability from enrollment to 3-month follow-up in perspectives on planning. Among participants choosing delivery, we found a significant shift toward more favorable planning perspectives at follow-up (from unplanned to ambivalent or planned, and ambivalent to planned). Among individuals choosing abortion, we found a significant shift toward less favorable planning perspectives at follow-up (from planned to ambivalently planned or unplanned, or ambivalently planned to unplanned).

These shifts in planning perspectives that align with pregnancy decision making are similar to subgroup analyses from the Turnaway Study, a 5-year prospective study of nearly 1000 pregnant people seeking abortion. In a subgroup analysis, they identified subsequent new pregnancies in 143 participants between 2008 and 2010 in the United States, and found participants who chose delivery had a favorable shift in planning (LMUP score increase of 2.2 at follow-up, indicating more planned), while participants who chose abortion demonstrated a shift toward less planned (LMUP score decrease of 0.7).^31^ Although the framework of the Turnaway Study as a longitudinal prospective study allows it be compared to our study, it has notable differences in methodology compared to our study.

In the Turnaway Study, all participants were asked every 6 months about the intendedness of any new pregnancy. Those who did not have a new pregnancy were asked about their prospective plans to become pregnant and those who had a pregnancy occurring since the previous assessment were asked to report retrospectively on that pregnancy's planning. In addition to this difference in methodology, the subgroup analysis of the Turnaway Study is characterized by a prolonged interval of assessment (6 months) compared to our study interval (3 months), and limited data are available on longitudinal analyses of intention in individuals who choose delivery.

In addition, we identified a shift from enrollment to 3-month follow-up in measures of happiness among participants who experienced miscarriage. This group demonstrated a shift in happiness (from happy to ambivalent or unhappy, or ambivalent to unhappy), findings that may represent a coping response to a pregnancy ending in miscarriage.^40,41^ However, these findings by pregnancy outcome are exploratory and limited by small sample size.

Our study uniquely examined the longitudinal assessment of six measures of preconception and postconception pregnancy contexts from early pregnancy to 3-month follow-up, included assessments of ambivalence to better understand the complexities of participants' pregnancy perceptions, and analyzed these context measures by pregnancy outcomes of delivery, abortion, and miscarriage. Prior studies are limited by an assessment of only one pregnancy context (focusing on preconception intention or planning), retrospective analyses, prolonged intervals between assessments, dichotomous pregnancy perspectives (intended or unintended without assessment of ambivalence), and, in many cases, singular pregnancy outcomes (pregnancies resulting in abortion or delivery only).^26,27,31,33^

Previous studies of the LMUP have analyzed it from preconception to postconception, ranging from 1 month before pregnancy to postpartum,^27,31^ but have not examined data this early in pregnancy or evaluated individual pregnancy context measures. In addition, our finding that pregnancy desire decreases at the 3-month follow-up among individuals choosing delivery warrants further evaluation using larger studies to investigate this finding. Prior studies, including a previous analysis of this study's cohort, found a correlation between undesired pregnancies and elevated Edinburgh Depression Screens, highlighting the importance of assessing pregnancy desire to identify patients who may benefit from early screening and intervention.^15,42^

While our study involved a diverse sample, it is limited by data collection in a single urban area. A majority of participants initially presented with an unintended pregnancy (56.2%), which is higher than national estimates of 45%.^1^ However, this rate may be explained by our cohort's demographics: A majority of our sample reported unemployment, limited education, and Black or Hispanic race/ethnicity—all factors that have been associated with higher unintended pregnancy rates.^1^

Our findings may be limited by not reassessing pregnancy contexts at the time of each pregnancy outcome (delivery, abortion, or miscarriage). In addition, although our study assesses pregnant people early in the first trimester (median gestational age 7 weeks and 3 days), we do not assess perspectives before or at the moment of a new conception; thus, we acknowledge these prepregnancy contexts are being measured retrospectively, although much earlier in pregnancy than previous studies. Finally, our relatively small sample size did not provide adequate power to assess pregnancy contexts by participants' sociodemographic characteristics. Larger studies with adequate power are needed to validate these findings.

Our findings that five of six pregnancy contexts remained unchanged from early pregnancy to 3-month follow-up among our overall sample point to a certainty in individuals' pregnancy perspectives. Participants in our study show stability in their assessments of pregnancy intention, wantedness, planning, timing, and happiness during the critical first and second trimester period, in which reproductive decision-making often takes place.

These findings may have implications for a majority of states in which abortion is legal, but mandatory waiting periods of 24–72 hours between abortion counseling and an abortion procedure may impose obstacles. These states often use the justification that pregnant individuals need adequate time to consider their pregnancy and weigh their options. However our exploratory data show that minimal change is observed over a much longer period of 3 months, which may counter this flawed legal rationale.^43,44^ Our study finds early pregnancy contexts are unchanged overall, emphasizing the reliability of individuals' assessments of their pregnancy and reproductive choice.^43,44^

## Conclusions

In a longitudinal assessment of multidimensional pregnancy contexts among a diverse cohort, participants demonstrated stability in most pregnancy perspectives from early pregnancy to 3-month follow-up. These findings illustrate the consistency of individuals' pregnancy perspectives in the first and second trimester and support pregnant individuals in making timely, individualized decisions for health care, including abortion care.

## Supplementary Material

Supplemental data

Supplemental data

Supplemental data

Supplemental data

Supplemental data

## Abbreviations Used

GED: general educational development
IQR: interquartile range
LMUP: London Measure of Unplanned Pregnancy
NIH: National Institutes of Health
NSFG: National Survey of Family Growth
PRAMS: Pregnancy Risk Assessment Monitoring System
