# Supplementary material for: A Multidimensional and Longitudinal Exploratory Study of the Stability of Pregnancy Contexts in the United States
Source: Womens Health Rep (New Rochelle). 2024 Mar 12;5(1):211–22. doi: 10.1089/whr.2024.0008 (PMC10956533; doi:10.1089/whr.2024.0008)
Supplement: Supplemental data [file Suppl_TableS1.docx]

**Supplementary Material**

**Table S1.** Description of pregnancy context measures

| **Pregnancy context** | **Question and response options** | **Category** |
| --- | --- | --- |
| *Preconception contexts* | | |
| Intention | Just before I became pregnant:^†^  I intended to get pregnant  My intentions kept changing  I did not intend to get pregnant | Favorable  Ambivalent  Unfavorable |
| Wantedness | Just before I became pregnant:^†^  I wanted to have a baby  I had mixed feelings about having a baby  I did not want to have a baby | Favorable  Ambivalent  Unfavorable |
| Planning | Based on the London Measure of Unplanned Pregnancy (LMUP)^27^:  Planned (scores 0-3)  Ambivalent (scores 4-9)  Unplanned (scores 10-12) | Favorable  Ambivalent  Unfavorable |
| *Postconception contexts* | | |
| Timing | In terms of becoming a mother (for the first time or again), I feel that my pregnancy happened at the:^†^  Right time  Okay but not quite right time  Wrong time | Favorable  Ambivalent  Unfavorable |
| Desire | Is this pregnancy desired?  Yes  Not sure  No | Favorable  Ambivalent  Unfavorable |
| Happiness | Rate how happy or unhappy you felt when you found out you were pregnant:  Very or somewhat happy  Neither happy nor unhappy/don’t know  Very or somewhat unhappy | Favorable  Ambivalent  Unfavorable |

^†^Based on individual elements from the LMUP.^33^ Table adapted from Lundsberg et al.^5^
