# Supplementary material for: A Multidimensional and Longitudinal Exploratory Study of the Stability of Pregnancy Contexts in the United States
Source: Womens Health Rep (New Rochelle). 2024 Mar 12;5(1):211–22. doi: 10.1089/whr.2024.0008 (PMC10956533; doi:10.1089/whr.2024.0008)
Supplement: Supplemental data [file Suppl_TableS2.docx]

**Supplementary Material**

**Table S2.** London Measure of Unplanned Pregnancy (LMUP).^37^ Adapted from Barrett et al 2004.

| **Question** | **Response** | **Score** |
| --- | --- | --- |
| In the month that I became pregnant: | I/we were not using contraception  I/we were using contraception, but not on every occasion  I/we always used contraception, but knew that the method had failed at least once  I/we always used contraception | 2  1  1  0 |
| In terms of becoming a mother (first time or again), I feel that my pregnancy happened at the: | Right time  OK, but not quite right time  Wrong time | 2  1  0 |
| Just before I became pregnant: | I intended to get pregnant  My intentions kept changing  I did not intend to get pregnant | 2  1  0 |
| Just before I became pregnant: | I wanted to have a baby  I had mixed feelings about having a baby  I did not want to have a baby | 2  1  0 |
| Before I became pregnant: | My partner and I had agreed that we would like me to get pregnant  My partner and I had discussed having children together, but hadn’t agreed for me to get pregnant  We never discussed having children together | 2  1  0 |
| Before you became pregnant, did you do anything to improve your health in preparation for pregnancy?  (tick all that apply) | Took folic acid  Stopped or cut down smoking  Stopped or cut down drinking alcohol  Ate more healthily  Sought medical/health advice  Took some other action (please describe)  I did not do any of the above before my pregnancy | 2 = ≥2 actions  1 = 1 action  0 |
| **Total score (range)** |  | **0-12** |
