# Supplementary material for: A Multidimensional and Longitudinal Exploratory Study of the Stability of Pregnancy Contexts in the United States
Source: Womens Health Rep (New Rochelle). 2024 Mar 12;5(1):211–22. doi: 10.1089/whr.2024.0008 (PMC10956533; doi:10.1089/whr.2024.0008)
Supplement: Supplemental data [file Suppl_TableS3.docx]

**Supplementary Material**

**Table S3.** Pregnancy contexts by outcome: delivery, N = 68

| **Pregnancy Context** | **Enrollment Responses, n (%)** | | | **Follow-up Responses, n (%)** | | |  |
| --- | --- | --- | --- | --- | --- | --- | --- |
|  | **Favorable** | **Unfavorable** | **Ambivalent** | **Favorable** | **Unfavorable** | **Ambivalent** | **P-value** |
| *Pre-conception* |  | | |  |  |  |  |
| Intention | 25 (36.8%) | 30 (44.1%) | 13 (19.1%) | 30 (44.1%) | 28 (41.2%) | 10 (14.7%) | 0.33 |
| Wantedness | 31 (45.6%) | 14 (20.6%) | 23 (33.9%) | 33 (48.5%) | 12 (17.7%) | 23 (33.8%) | 0.69 |
| Planning | 25 (36.7%) | 4 (5.9%) | 39 (57.4%) | 23 (33.8%) | 6 (8.8%) | 39 (57.4%) | < 0.01 |
| *Post-conception* |  | | |  |  |  |  |
| Timing | 34 (50.0%) | 4 (5.9%) | 30 (44.1%) | 34 (50.0%) | 3 (4.4%) | 31 (45.6%) | 0.81 |
| Desirability | 50 (73.5%) | 2 (7.4%) | 13 (19.1%) | 35 (51.5%) | 16 (23.5%) | 17 (25.0%) | < 0.01 |
| Happiness | 55 (80.9%) | 2 (2.9%) | 11 (16.1%) | 54 (79.4%) | 4 (5.9%) | 10 (14.7%) | 0.73 |
