# Supplementary material for: A Multidimensional and Longitudinal Exploratory Study of the Stability of Pregnancy Contexts in the United States
Source: Womens Health Rep (New Rochelle). 2024 Mar 12;5(1):211–22. doi: 10.1089/whr.2024.0008 (PMC10956533; doi:10.1089/whr.2024.0008)
Supplement: Supplemental data [file Suppl_TableS4.docx]

**Supplementary Material**

**Table S4.** Pregnancy contexts by outcome: abortion, N = 34

| **Pregnancy Context** | **Enrollment Responses, n (%)** | | | **Follow-up Responses, n (%)** | | |  |
| --- | --- | --- | --- | --- | --- | --- | --- |
|  | **Favorable** | **Unfavorable** | **Ambivalent** | **Favorable** | **Unfavorable** | **Ambivalent** | **P-value** |
| *Pre-conception* |  | | |  |  |  |  |
| Intention | 4 (11.8%) | 27 (79.4%) | 3 (8.8%) | 2 (5.9%) | 29 (85.3%) | 3 (8.8%) | 0.38 |
| Wantedness | 3 (8.8%) | 20 (58.8%) | 11 (32.4%) | 5 (14.7%) | 19 (55.9%) | 10 (29.4%) | 0.73 |
| Planning | 4 (11.8%) | 20 (58.8%) | 10 (29.4%) | 2 (5.9%) | 16 (47.05%) | 16 (47.05%) | < 0.01 |
| *Post-conception* |  | | |  |  |  |  |
| Timing | 2 (5.9%) | 22 (64.7%) | 10 (29.4%) | 2 (5.9%) | 19 (55.9%) | 13 (38.2%) | 0.45 |
| Desirability | 5 (14.7%) | 21 (61.8%) | 8 (23.5%) | 4 (11.8%) | 19 (55.9%) | 11 (32.3%) | > 0.99 |
| Happiness | 11 (32.4%) | 12 (35.3%) | 11 (32.4%) | 9 (26.5%) | 11 (32.3%) | 14 (41.2%) | 0.80 |
