# Supplementary material for: A Multidimensional and Longitudinal Exploratory Study of the Stability of Pregnancy Contexts in the United States
Source: Womens Health Rep (New Rochelle). 2024 Mar 12;5(1):211–22. doi: 10.1089/whr.2024.0008 (PMC10956533; doi:10.1089/whr.2024.0008)
Supplement: Supplemental data [file Suppl_TableS5.docx]

**Supplementary Material**

**Table S5.** Pregnancy contexts by outcome: miscarriage, N = 18

| **Pregnancy Context** | **Enrollment Responses, n (%)** | | | **Follow-up Responses, n (%)** | | |  |
| --- | --- | --- | --- | --- | --- | --- | --- |
|  | **Favorable** | **Unfavorable** | **Ambivalent** | **Favorable** | **Unfavorable** | **Ambivalent** | **P-value** |
| *Pre-conception* |  | | |  |  |  |  |
| Intention | 3 (16.7%) | 11 (61.1%) | 4 (22.2%) | 4 (22.2%) | 14 (77.8%) | 0 (0.0%) | > 0.99 |
| Wantedness | 4 (22.2%) | 8 (44.5%) | 6 (33.3%) | 7 (38.9%) | 5 (27.8%) | 6 (33.3%) | 0.07 |
| Planning | 2 (11.1%) | 5 (27.8%) | 11 (61.1%) | 3 (16.7%) | 5 (27.8%) | 10 (55.5%) | 0.73 |
| *Post-conception* |  | | |  |  |  |  |
| Timing | 8 (44.4%) | 5 (27.8%) | 5 (27.8%) | 5 (27.8%) | 7 (38.9%) | 6 (33.3%) | 0.13 |
| Desirability | 6 (33.3%) | 7 (38.9%) | 5 (27.8%) | 7 (38.9%) | 4 (22.2%) | 7 (38.9%) | 0.69 |
| Happiness | 12 (66.7%) | 2 (11.1%) | 4 (22.2%) | 9 (50.0%) | 9 (50.0%) | 0 (0.0%) | 0.02 |
